# Circadian Rhythm Alterations May be Related to Impaired Resilience, Emotional Dysregulation and to the Severity of Mood Features in Bipolar I and II Disorders

**DOI:** 10.1192/j.eurpsy.2023.830

**Published:** 2023-07-19

**Authors:** L. Palagini, A. Gmignani, L. Grassi, P. A. Geoffroy

**Affiliations:** 1 University of Ferrara, ferrara; 2 University of Pisa, Pisa, Italy; 3 University of Paris, Paris, France

## Abstract

**Introduction:**

Recent theories hypothesized that chonobiological dis-rhythmicity might contribute to Bipolar disorders (BD) by dysregulating most of the systems involved in mood, stress and emotion regulation. In particular, the key role of sleep in regulating stress system and emotions has been hypothesized. Among other important factors contributing to BD the stress vulnerability/resilience dimension may play a key role. In particular low resilience has been associated with a dysregulation in emotions and stress response possibly involved in psychopathological process of BDs

**Objectives:**

The study aimed to investigate the possible impact of resilience and emotion dysregulation on the clinical manifestations of bipolar disorders (BDs) focusing on the possible role of circadian rhythm alterations.

**Methods:**

A sample of 197 inpatients suffering from BD of type I (BDI) or II (BDII) were assessed during a major depressive episode using the Structural Clinical Interview for DSM-5 (SCID-5), the Beck Depression Inventory-II (BDI-II), the Young Mania Rating Scale (YMRS), Resilience Scale for Adults (RSA), Biological Rhythms Interview of Assessment in Neuropsychiatry (BRIAN), Difficulties in Emotion Regulation Scale (DERS) and the Scale for Suicide Ideation (SSI). Participants with or without circadian rhythm disturbances as measured with Biological Rhythms Interview of Assessment in Neuropsychiatry (BRIAN), were compared; regression and mediation analyses were computed.

**Results:**

Participants with circadian rhythms disturbances showed a greater severity of depressive symptoms, of suicidal risk, lower resilience and more disturbances in emotion regulation including impulsivity and regulatory strategies. The logistic regression revealed that circadian rhythm disturbances was related to depressive symptoms (O.R. 4.0), suicidal risk (OR 2.51), emotion dysregulation (OR 2.28) and low resilience (OR 2.72). At the mediation analyses, circadian rhythm alterations showed an indirect effect on depressive symptoms by impairing resilience (Z= 3.17, p=0.0014)/ emotional regulation (Z= 4.36, p<0.001) and on suicidal risk by affecting resilience (Z= 2.00, p=0.045) and favoring impulsivity (Z= 2.14, p=0.032).

**Conclusions:**

The present findings may show that circadian rhythm alterations might play a key role in BD manifestations, as being correlated with more severe clinical presentations of depressive symptoms, suicidal risk, impaired resilience and emotional dysregulation. Addressing circadian rhythm alterations might potentially promote resilience and emotion regulation hence improving mood symptoms and suicidal risk in BDs.

**Disclosure of Interest:**

None Declared

